# The In Vitro Anti-amyloidogenic Activity of the Mediterranean Red Seaweed *Halopithys Incurva*

**DOI:** 10.3390/ph13080185

**Published:** 2020-08-07

**Authors:** Marzia Vasarri, Matteo Ramazzotti, Bruno Tiribilli, Emanuela Barletta, Carlo Pretti, Nadia Mulinacci, Donatella Degl’Innocenti

**Affiliations:** 1 Department of Experimental and Clinical Biomedical Sciences, University of Florence, Viale Morgagni 50, 50134 Florence, Italy; marzia.vasarri@unifi.it (M.V.); matteo.ramazzotti@unifi.it (M.R.); emanuela.barletta@unifi.it (E.B.); 2 Institute for Complex Systems-National Research Council (ISC-CNR), Via Madonna del piano 10, 50019 Sesto Fiorentino, Florence, Italy; bruno.tiribilli@isc.cnr.it; 3 Department of Veterinary Sciences, University of Pisa, Viale delle Piagge 2, 56124 Pisa, Italy; carlo.pretti@unipi.it; 4 Interuniversity Center of Marine Biology and Applied Ecology “G. Bacci” (CIBM), Viale N. Sauro 4, 57128 Livorno, Italy; 5 Department of NEUROFARBA, Nutraceutical section, University of Florence, Via Ugo Schiff 6, 50019 Sesto Fiorentino, Italy; nadia.mulinacci@unifi.it

**Keywords:** *Halophytis incurva*, red seaweed, amyloid aggregation, HEWL, anti-amyloidogenic

## Abstract

Neurodegenerative diseases are generally characterized by the presence of neurotoxic amyloid aggregates underlying progressive neuronal death. Since ancient times, natural compounds have been used as curative agents for human health. Amyloid research is constantly looking for safe natural molecules capable of blocking toxic amyloid aggregates’ formation. From the marine environment, seaweeds are recognized as rich reservoirs of molecules with multiple bioactivities, including the anti-amyloidogenic activity. Here, hydroalcoholic extracts of two seasonal samples of the Mediterranean red seaweed *Halophytis incurva* (HIEs) were characterized by the HPLC-DAD-MS analysis. The *H. incurva* anti-amyloidogenic role was explored by incubating both HIEs with hen egg white lysozyme (HEWL), a well-known protein model widely used in amyloid aggregation experiments. The aggregation kinetics and morphological analysis of amyloid aggregates were performed by ThT and AFM analysis, respectively, while their cytotoxicity on SH-SY5Y human neuroblastoma cells was examined by MTT assay. HIEs showed a different efficacy, probably dependent on their metabolic composition, both in inhibiting amyloid fibrillation and in obtaining short and less toxic pre-fibrillary aggregates. Overall, this work sheds light, for the first time, on a Mediterranean red seaweed as a promising renewable resource of bioactive compounds, potentially useful in preventing the formation of toxic amyloid aggregates.

## 1. Introduction

Neurodegenerative diseases affect millions of people around the world with a growing prevalence along with rapidly aging populations. They are characterized by the progressive loss of neurons due to the deposition of proteins with aberrant physical–chemical properties in the brain and peripheral organs. With a relatively long course of the disease and high costs for medical care, neurodisorders have become a huge burden on families and society and a serious public health challenge [1]. Currently, the available treatments are only symptomatic and there are still no resolutive therapies to slow the progression of neuronal degeneration. In this regard, a collaborative network has been created between medical centers, research institutes and highly qualified specialists to find innovative, sustainable and effective paths with the aim of providing new, optimal and safer approaches to prevent the onset of such debilitating diseases [2]. Amyloid fibrils’ formation and their subsequent deposition in different organs and tissues are crucial events for the onset of neurodegenerative diseases [3]. Indeed, the progressive accumulation of toxic amyloid aggregates triggers a cascade of harmful cellular events until progressive neuronal death [4]. Therefore, scientific research is constantly looking for novel natural molecules capable of blocking the formation of toxic amyloid fibrils from different proteins [5,6]. Interestingly, the molecular modeling and chemical synthesis of effective and specific drugs, potentially useful for the prevention and/or treatment of various human diseases, has been based on the unusual and specific chemical structure of various marine natural bioactive compounds [7]. Indeed, the huge number of marine organisms and their unique chemical diversity have long encouraged researchers to explore the marine environment in search of powerful molecules with anti-amyloid properties [8,9]. Among marine organisms, seaweeds have long been considered an abundant and renewable source of structurally different and biologically active compounds with interesting pharmaceutical and biomedical potential for various chronic diseases, including neurodegenerative disease [10]. Even before the development of modern medicine, people relied on a broad spectrum of natural remedies for the treatment of central nervous-system-related diseases, and for this purpose many species of seaweeds have long been used in food diets and traditional remedies in Eastern countries, but also in Europe and America. Based on this knowledge, seaweeds are considered optimal candidates both in the food and pharmaceutical fields for the development of new pharmaceutical products or powerful bioactive substances for the prevention of neurodegenerative disorders [10]. *Halopithys incurva* (HIE) (Hudson) Batter (a.k.a., Red Sea Pine) is a slow-growing, sun-adapted perennial to annual small filamentous red seaweed classified as *Rhodophyta, Ceramiales, Rhodomelaceae*. It is particularly abundant in the Mediterranean Sea, generally located on rocky substrates in a not very sunny area at a depth ranging from a few centimeters to about ten meters up to a maximum of twenty meters (see www.algaebase.org, species id 116, for a detailed distribution survey). In the past, *H. incurva* has been studied in terms of adaptive response to seasonal changes [11] and, above all, for its richness in halogenated secondary metabolites [12,13], in particular polyphenols with high antioxidant power [14]. However, little is known about the bioactive properties of *H. incurva* seaweed. Some studies have attributed extracts’ effective antibacterial, antiviral, antiplasmodial, antiprotozoal, antimycobacterial and strong anti-inflammatory properties to *H. incurva* [15,16,17]. Furthermore, *H. incurva* has exhibited polysaccharide-mediated immunomodulatory properties [18] and antitumor and antioxidant potential on HT-29 human colorectal adenocarcinoma cells [19]. To the best of our knowledge, seaweeds rich in phenolic compounds have already been studied as a source of compounds with anti-amyloidogenic properties potentially useful in the prevention and/or treatment of amyloidosis [20,21,22]. Furthermore, a large body of the literature reports that plant polyphenols are of great benefit to general human health [23]. Several phenolic compounds, with antioxidant properties, were also demonstrated to be able to inhibit the amyloid fibrils’ formation, disaggregate preformed fibrils or moderate their cytotoxicity with mechanism of action apart from its antioxidant role [24,25,26]. Lysozyme, a 130 residue bacteriolytic enzyme, is commonly used as a valid model protein for the study of amyloid aggregation. There is no direct correlation between wildtype lysozyme and amyloid diseases. However, systemic amyloidosis in humans can be related to several naturally occurring single point mutations of lysozyme, i.e., Ile56Thr, Phe57Ile, Trp64Arg and Asp67His [27]. Moreover, it has been observed that wild type lysozyme from humans, horses or chickens, can form amyloid fibrils in vitro under suitable conditions [28]. In particular, high temperatures and acid conditions cause hen egg white lysozyme (HEWL) to form fragments of peptides, containing amino acid residues corresponding to those mutated in human family diseases. These fragments have a high tendency to form amyloid aggregates in a few days [29]. Hence, the HEWL amyloid model is widely used and accepted to study the mechanism of amyloid fibrils formation and its inhibition by small molecules [30]. Therefore, the effect of hydroalcoholic extracts from two different *H. incurva* seasonal samples on the HEWL amyloid aggregation was investigated in this work. In addition, the effect of *H. incurva* extracts on cytotoxicity, typically induced by HEWL fibrils, was verified on SH-SY5Y human neuroblastoma cells.

## 2. Results and Discussion

### 2.1. Extraction Procedure and Biochemical Composition of the H. Incurva Extracts

**Figure 1:**
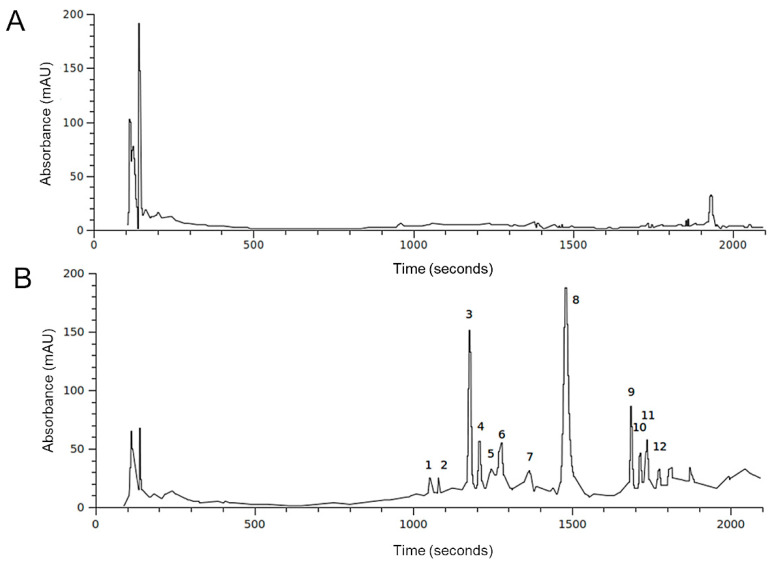
HPLC profiles at 280 nm of the *H. incurva* ethanol extracts. The peaks in HIE/m (**A**) and HIE/n (**B**) are labeled according to Table 2.

The hydroalcoholic extraction method from plant material has already been proven to be an effective method for recovering a significant quantity of hydrophilic compounds [31]. In this work, our hydroalcoholic extraction from the two different *H. incurva* seasonal samples allowed us to recover about 58 mg (~1.5%) and 520 mg (~13%) of dry extract, respectively from the May (m) and November (n) samples, starting from 4 g of dry seaweed (Table 1). The final yield in dry extract indicated a strong influence of the season on the composition of this red seaweed. The chromatographic analysis confirmed a substantial difference in the *H. incurva* metabolic content for the two seasonal states (spring and autumn). This result suggests that changes in the *H. incurva* growth conditions, driven by seasonal variations, have a strong influence on the composition of its secondary metabolites. Hence, seasonality is undoubtedly a relevant factor to consider when collecting the sample, since a different content of secondary metabolites may reflect differences in the seaweed bioactive properties and in their effectiveness. The phenolic and carbohydrate content of HIEs were measured, respectively, by Folin–Ciocalteau and phenol-sulfuric acid assays. As shown in Table 1, HIE/n was found to be almost ten times more abundant in hydrophilic compounds (13 ± 1%) than HIE/m (1.45 ± 0.1%). Despite this, HIE/n contained a lower amount of total polyphenols (37.4 ± 0.8 mg/g of dry extract) compared to HIE/m (69.3 ± 1.1 mg/g of dry extract). The difference in the metabolic composition was even more evident when the carbohydrate content was measured with the phenol-sulfuric acid assay, indicating values of 315 ± 31 and 80.6 ± 3.8 mg/g of dry extract for HIE/m and HIE/n, respectively. Since phenolic compounds and carbohydrates are known to act as scavengers for free radicals and antioxidants in general, we tested HIEs with the DPPH and FRAP assays. The antioxidant activity of HIE/m (137.4 ± 14.7 mg/g of dry extract) was five times higher than that of HIE/n (24.8 ± 1.8 mg/g of dry extract). The difference in the radical scavenging activity was notably less pronounced between the two seasonal samples, however, it was lower in HIE/m (45.6 ± 3.6 mg/g of dry extract) than in HIE/n (82.6 ± 0.5 mg/g of dry extract). Overall, it was obtained that HIE/m was more enriched in polyphenols (about twice); this result perfectly agrees with the chemical profiles obtained by HPLC-DAD focused on highlighting the main secondary metabolites in the ethanol extracts (Figure 1). Carbohydrate content in HIE/m was about four times higher than in HIE/n. Since a similar proportion (approximately five times) was observed for the antioxidant activity, it can be assumed that the carbohydrate fraction of HIE/m may be involved in the antioxidant power. However, simple carbohydrates are not capable of functioning as antioxidants themselves, but it is necessary that they possess one or more functional groups [32]. In this regard, it could be hypothesized that the HIE/m is enriched in functionalized carbohydrates, as already proven in similar red algae [33].

### 2.2. HPLC-DAD-MS Analysis of H. Incurva Extracts

To investigate the HIEs’ metabolic composition, a first characterization on HIE/m and HIE/n was performed with HPLC-DAD-MS analysis. The reference wavelength of 280 nm was selected as the most appropriate for showing the profile of the extracts. Figure 1 compares the sample profiles obtained from the same initial amount of H. incurve, showing strong differences in terms of detected secondary metabolites.

**Figure 2:**
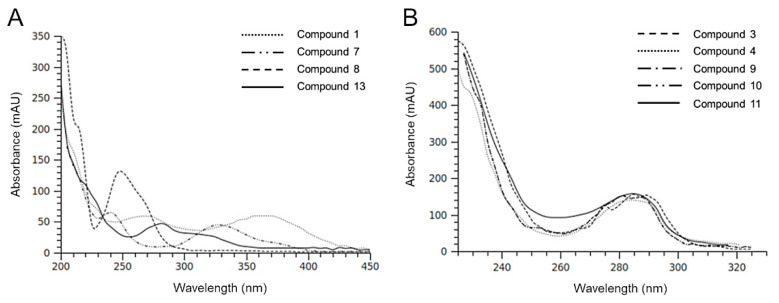
UV-Vis spectra of the main compounds detected in HIE/n. In (**A**) and (**B**) the numbering scheme reflects that reported in Figure 1 and Table 2. The UV-Vis spectra of **9** and **10** (**B**) are exactly overlapped.

In accordance with data in Table 1, HIE/n exhibited a greater number of phenolic compounds and a lower content of a very polar fraction (rt range 100–200 sec) compared to HIE/m. Most of the compounds detected had similar UV-Vis spectra (Figure 2) indicating a possible presence of a phenolic nucleus in their structures. Among these, compound **1,** with a different chromophore having a maximum at 370 nm (Figure 2A) is, in part, responsible of the yellow/brown color of the HIE/n extract.

In order to verify the presence of brominated compounds previously identified in the red seaweed *H. incurva* [34] and in other seaweeds [35,36], mass spectra of HIE/n sample were recorded in negative ionization mode, obtaining the results summarized in Table 2. 

**Figure 3:**
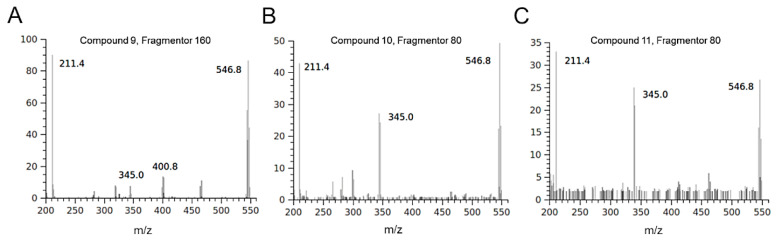
Mass spectra of isobaric halogenophenols detected in HIE/n recorded in negative ionization mode. The compounds showed a different fragmentation pattern depending on the applied energy: 160 V for compound **9** (**A**) and 80 V for compounds **10** (**B**) and **11** (**C**).

The isotopic distribution of bromine ions allows the mass spectra of molecules containing these atoms to show diagnostic clusters [37]. Some molecules detected in the HIE/n sample showed fragmentation patterns indicating the presence of bromine atoms. Compounds **3** and **4** were tentatively identified as two isobaric monobromopnehonls with a typical cluster for the [M-H]^–^ ions at m/z 347 and m/z 345, both at the same intensity, and the species at m/z 267 produced by the loss of 80 Da attributable to a bromine atom. Compound **8** can be tentatively identified as 3 bromo, 4,5 dihydroxyphenyl methanol, previously detected in the red algae [36]. The three isobaric compounds **9**, **10** and **11** showed the same UV-Vis spectrum and a typical fragmentation pattern, which suggested the presence of two bromine atoms in their structure (Figure 3). Several monobromo- and dibromophenols have been previously detected in red and green seaweeds [35,36]. As for compound **9**, the maximum intensity of the [M-H]^–^ ion at 547 m/z was obtained with fragmentor at 160 V, conversely, for compounds **10** and **11** the maximum intensity of [M-H] ^–^ ion was reached by applying a lower energy for fragmentation (80 V)**.** For all the isobars, the ion clusters at 465 and 467 m/z indicated the loss of a bromine atom and the species at 401 and 403 m/z were attributable to a moiety containing a bromine atom that originated the ion at 321 m/z after the loss of 80 Da. All the mass spectra of the three isobars showed the presence of a non-halogenated fragment at m/z 211.

The results from HPLC-DAD-MS noticed strong difference in terms of metabolic composition between the *H. incurva* extracts obtained from the two seasonal samples. This substantial difference reflects a difference in the role of the two extracts on the amyloid aggregation process as demonstrated in subsequent experiments on the formation of amyloid aggregates in the presence of HIEs.

### 2.3. Effect of H. Incurva Extracts on HEWL Amyloid Fibril Formation

**Figure 4:**
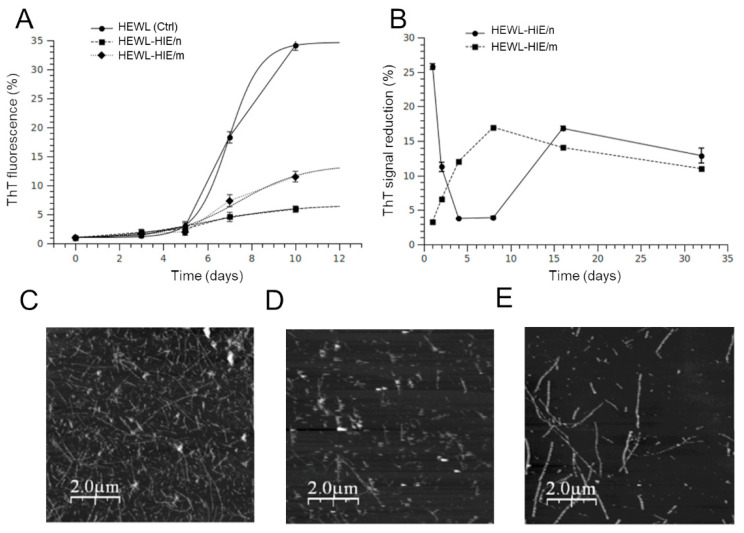
Effect of the HIEs on the HEWL amyloid aggregates formation. (**A**) Kinetic plots followed by ThT fluorescence signals. Plots report the ThT fluorescence signals at different times with respect to that at 0 days. (**B**) ThT competition assay. Preformed HEWL fibrils were incubated with ThT dye in the presence of HIE/m or HIE/n. (**C**–**E**) Representative AFM morphological analysis images showing the quantitative and qualitative differences in the HEWL fibrils’ formation at 10 days of incubation of HEWL alone (**C**) or in the presence of HIE/m (**D**) or HIE/n (**E**).

Since amyloid aggregation is considered the initiator of events leading to neurotoxicity and clinical symptoms of amyloidosis diseases, the search for effective anti-amyloidogenic agents has become an important strategy in amyloid research [38]. To date, blocking the formation of amyloid aggregates through direct inhibition of the self-assembly process with small molecules has been proposed as a potentially valid strategy for the development of novel approaches in the field of neurodegeneration prevention [39]. Among various protein model systems, HEWL is widely used to examine the structural and mechanistic principles underlying the amyloid aggregation. Indeed, the formation of HEWL fibrils shares many similarities, in terms of aggregation kinetics and morphology of the aggregated species, with the fibrillation of proteins involved in the pathogenesis of neurodegenerative diseases, including the amyloid Alzheimer Aβ1–40 peptide [40,41]. In addition, HEWL represents an attractive and biomedi·ally relevant model system for the characterization of intermediate amyloid species since the aggregation rate can be controlled, unlike other model systems, through simple experimental interventions, such as changes in the pH or temperature of the aggregation solution. Furthermore, the aggregation kinetics, the lag phase or the fibrils morphology are sometimes altered in many experimental amyloid models by copper or iron ions, even in traces. This phenomenon never occurred for the HEWL amyloid model. Our HIEs were particularly rich in polyphenols, widely documented for their metal chelation properties [42]. Consequently, the choice to use HEWL as a model system to study the role of HIEs role on amyloid fibrillation allowed us to separate the effect of metal ions on the aggregation process and establish a direct connection between HIE compounds and their role, overall, on the amyloid aggregation process [43]. In order to monitor the growth of HEWL fibrils, the ThT fluorescence assay was performed. The aggregation kinetics of HEWL and HEWL incubated with HIE/m or HIE/n, illustrated in Figure 4A, showed that both HIEs had a strong impact on the amyloid aggregation process. In fact, the ThT fluorescence intensity of HEWL-HIE/m and HEWL-HIE/n was significantly reduced by about 3 and 7 times, respectively, compared to that of HEWL grown in the absence of HIEs. At 10 days of incubation, the HEWL aggregation process in the presence of HIEs reached a plateau phase. This event suggests that the presence of HIEs considerably slows down the amyloid aggregation process, making it very difficult to form, at longer times (over 10 days), amyloid aggregates numerically and or structurally comparable to those obtained in the absence of HIEs. Therefore, it is presumed that HEWL-HIE aggregates stabilize in the conformation achieved at 10 days of incubation. Although the HIEs’ inhibitory role on the HEWL fibrils’ formation was decidedly evident, the ThT analysis results could be altered by a possible interference of heterologous compounds present in the extracts, such as polyphenols, with ThT dye [44]. To rule out the possibility that the observed fluorescence intensity reduction could be attributed to a competition between ThT dye and some HIEs compounds, each HIE was added to preformed HEWL fibrils and ThT fluorescence signals were immediately measured. It was shown that only a fraction of the observed inhibition could be attributed to interference or competition phenomena, reinforcing our findings on the ability of HIEs to block the formation of HEWL amyloid fibrils (Figure 4B). Furthermore, the lack of significant changes in the early stage of HEWL amyloid fibrillation process supports the idea that HIEs considerably slow down the formation of amyloid fibrils. With the aim of defining the nature of the HEWL-aggregated species, formed in the absence or presence of HIEs, their morphology was examined using AFM analysis. As illustrated in Figure 4C, the 10-day aggregation (corresponding to the plateau phase in the aggregation process, see Figure 4A) of HEWL in the absence of HIEs led to the formation of well-defined mature fibrils with typical long and unbranched morphology. On the other hand, when HEWL was incubated with HIEs, the total load of formed fibrillar structures was significantly reduced and a mixture of shorter fibrillar assemblies along with oligomeric and/or amorphous species appeared (Figure 4D,E). These results agree perfectly with those of the ThT assay on the ability of HIEs to inhibit the formation of HEWL amyloid fibrils. Furthermore, it was curiously observed that that HEWL-HIE/m aggregates were shorter than HEWL-HIE/n (Figure 4D,E). These results suggest that the two HIEs acted differently on the amyloid aggregation process, favoring the formation of qualitatively different aggregated species. The different metabolic composition of the two seasonal samples probably had a decisive influence on the HIEs’ role in amyloid fibrillation. It is of reasonable importance to underline that a phytocomplex generally shows a better biological efficacy due to the synergistic action between its secondary metabolites with respect to the activity of the individual compounds. The high HIE content of polyphenols and carbohydrates, widely documented to play a crucial role in the amyloid aggregation process [23,45], suggests that the *H. incurva* anti-amyloidogenic role, here proven, is the result of the synergistic action of its secondary metabolites. Although this work sheds light for the first time on the anti-amyloidogenic role of a red Mediterranean seaweed, however its composition, rich in total phenols and carbohydrates, finds analogies with other seaweeds, with similar bioactivities, widespread in the Pacific and Indian Ocean countries, such as the red seaweed G. acerosa [22,46] or the brown seaweed P. gymnospora [47] which are endowed with anti-aggregating properties on the Aβ peptide. Taken together, our results strongly support *H. incurva* as a new potential source of bioactive compounds against amyloidosis.

### 2.4. Evaluation of HEWL Amyloid Aggregate-Induced Cytotoxicyty

**Figure 5:**
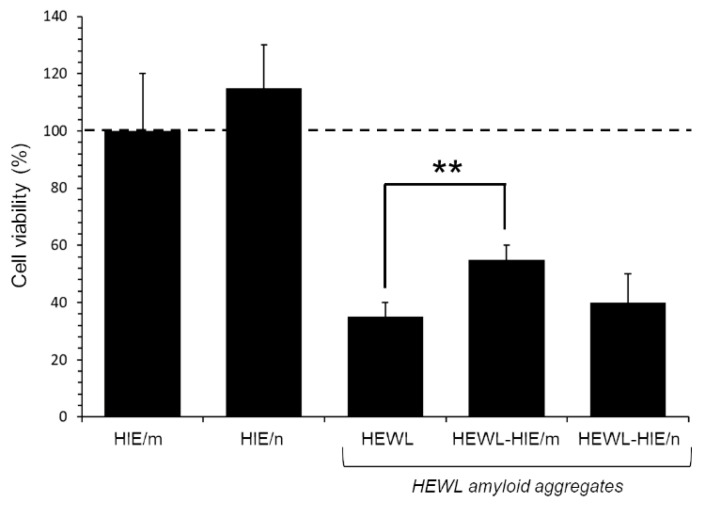
Effect of HIEs on cytotoxicity induced by HEWL aggregates on SH-SY5Y cells. Cells were treated for 48 h with HEWL aggregates formed for 4 days in the absence or presence of HIE/m or HIE/n. Data are reported as percentage values with respect to the untreated control cells (represented by the dotted line) and expressed as the mean ± standard deviation of at least three independent experiments of three independent extractions. Error bars represent standard deviations. Statistical significance was performed using T-test: **: *p*-Value < 0.01 vs. the untreated control cells.

To verify whether HIEs had an effect on the HEWL aggregate-induced cytotoxicity, as well as on the reduction in aggregate load, an MTT test was performed on SH-SY5Y human neuroblastoma cells, a cell line model widely used to test amyloid aggregate-induced neurotoxicity [43]. Specifically, cells were exposed for 48 h to HEWL aggregates (at 1:100 final dilution) matured for 4 days alone or in the presence of HIEs. As previously demonstrated by a kinetic analysis of cytotoxicity, the incubation time of 3 or 4 days proved to be the time spectrum in which the HEWL aggregates, under our experimental conditions, were more toxic, while the native HEWL showed no sign of cytotoxicity [43]. As illustrated in Figure 5, while HIEs alone proved safe for cells at the concentrations used in the test (0.11 and 1 μg/mL for HIE/m and HIE/n, respectively), the viability of cells treated with HEWL amyloid fibrils was significantly reduced by about 70 ± 5% compared to untreated control cells. Although a marked toxicity persisted, however, HEWL amorphous and unstructured fibrils obtained in the presence of HIE/m were remarkably less toxic than HEWL fibrils formed in the absence of HIE/m. Indeed, MTT assay results showed a significant 20 ± 2% recovery of viability in HEWL-HIE/m treated cells compared to HEWL treated cells. On the contrary, HIE/n was not able to mitigate the toxicity of HEWL aggregates, which therefore remained invariably toxic compared to those grown in the absence of HIEs. It is currently known for many amyloid-forming proteins by which monomers and full-length fibrils induce limited toxicity to neurons or other cells, while both non-fibrillary and small fibrillary aggregates are primarily responsible for amyloid toxicity [48]. Consequently, the identification of amyloid fibril growth inhibitors is considered an important goal in the research into amyloid toxicity prevention. In this regard, a huge body of the literature reports that molecules with peculiar structures are capable of interfering with the formation or lengthening of amyloid fibrils. Among these molecules are naturally occurring polyphenols [5,49,50,51], the hydrophobic groups of which are able to interfere with the amyloid aggregation process, also forming stable hydrogen bonds with the protein [5,30]. The literature discloses that polyphenols may have the ability to inhibit the formation of the first toxic oligomers favoring the formation of stable and non-toxic protofibrils preventing the evolution in other toxic amyloid structures. Furthermore, it is reported that polyphenols, if added to preformed fibrils, can be capable of neutralizing any toxic effect of soluble oligomer residues or other toxic aggregates [52]. As discussed above, the AFM analysis showed that the presence of HIE/m during HEWL aggregation leads to the formation of obviously shorter fibrillary assemblies along with oligomeric and/or amorphous species compared to those obtained in the presence of HIE/n. Curiously, the HEWL-HIE/m aggregates, despite their smaller size, exhibited a reduced cytotoxicity on SH-SY5Y cells, otherwise not proven for HEWL-HIE/n. Therefore, in light of these considerations, it can be assumed that HIE/m slowing down the amyloid aggregation process could promote the formation of less toxic oligomeric species for cells.

Considering the possible disproportion between the total load of toxic HEWL aggregates and the presumably low dose of protective molecules present in HIE/m, the significant reduction in the HEWL-HIE/m cytotoxicity should be considered promising in this pilot experiment. In our in vitro HEWL-based amyloid assay, we deliberately added a reduced amount of HIEs (1:50) to exalt possible important inhibitory properties against amyloid aggregation of hydrophilic *H. incurva* compounds. When cytotoxicity was assessed, HIEs as well as HEWL aggregates were further diluted 1:100. We emphasize that this extreme dilution of HIEs (1:5000 in cell medium) substantially excludes the contribution to neuroprotection against amyloid aggregate-driven injury of their inherent (see Table 1) antioxidant and radical scavenging activity [45]. Therefore, the positive impact of HIE/m must be assigned to a yet unexplored interference of its (inside) metabolites with the amyloid aggregation process. Nevertheless, the important antioxidant and radical scavenging activities present in HIEs, though probably disjointed from the anti-amyloidogenic activity, could contribute significantly to the possible beneficial properties of HIEs.

## 3. Materials and Methods

### 3.1. Materials and Reagents

Dulbecco’s modified Eagle’s medium (DMEM), Ham’s F-12 nutrient mixture, fetal bovine serum (FBS), L-glutamine, penicillin and streptomycin, 1-(4,5-dimethylthiazol-2-yl)-3,5-diphenyl formazan (MTT), α,α-Diphenyl-β-picrylhydrazyl (DPPH), 3-(2-Pyridyl)-5,6-diphenyl-1,2,4-triazine-4′,4′′-disulfonic acid sodium salt (Ferrozine™), Thioflavin T (ThT), Folin–Ciocalteau’s reagent, hen egg white lysozyme (HEWL), gallic acid, ascorbic acid, D-glucose and all other chemicals, analytical grade and HPLC grade solvents were purchased from Sigma Aldrich-Merck (Saint Louis, MO, USA). Disposable plastics were from Sarstedt (Nümbrecht, Germany).

### 3.2. H. Incurva Collection and Extraction

Field collection and identification of *H. incurva* samples were carried out by CIBM experienced personnel in May and November from the rocky substrates of the Tyrrhenian Sea (Livorno, Tuscany, Italy) at a depth of about 2–5 m. Immediately after harvesting, *H. incurva* seaweed was gently washed with bi-distilled water to remove salts, epibiota and other contaminants and dried overnight at 60 °C. Then, dried seaweed was minced and resuspended with 5 mL of EtOH/H2O (70:30 *v/v*) per gram of dry seaweed with the aim at collecting substantially hydrophilic compounds [31]. The extraction was carried out twice under continuous stirring at room temperature, the first overnight and the second for 6h. The *H. incurva* hydroalcoholic extract was separated from debris and mixed with n-hexane (1:1 ratio). The hydrophilic fraction, recovered in the lower phase, was dispensed in several batches and then dried using a UnivapoTM vacuum-spin concentrator (UniEquip, Planegg, München, Germany). Each batch (corresponding to 0.8 g of dry seaweed) was suspended in 2 mL of bi-distilled water prior to use. Hydroalcoholic extracts of *H. incurva* May and November samples were hereinafter called HIE/m and HIE/n, respectively.

### 3.3. Total Phenolic and Carbohydrate Content

The total phenolic (TP) and carbohydrate (TC) content of HIEs was determined according to the Folin–Ciocalteau’s and phenol–sulfuric acid methods, respectively, described in [31,53,54]. The TP and TC content was determined using gallic acid (0–10 μg) and D-glucose (0–50 μg), respectively, as reference.

### 3.4. Antioxidant and Radical Scavenging Assays

The antioxidant and radical scavenging activities of HIEs were determined according to the ferric-reducing/antioxidant power (FRAP) assay and DPPH assay, respectively, previously described [31,53,54]. Both bioactivities were measured using ascorbic acid (0.1 mg/mL) as a reference in the range of 0–4 μg.

### 3.5. HPLC-DAD-MS Analysis

The hydroalcoholic *H. incurva* extracts from the two seasonal samples were dried, and aliquots corresponding to 0.2 g of dried seaweed were dissolved using 0.5 mL of an ethanol/water mixture pH 3.2 by HCOOH 8:2. Then, samples were centrifuged at 12,000 rpm for 4 min and the supernatant was directly injected in HPLC. Analyses were carried out using a HP 1100L liquid chromatograph equipped with a DAD detector coupled to a HP 1100 MSD mass spectrometer with an API/electrospray interface (all from Agilent Technologies, Palo Alto, CA, USA). A 150 × 3.9 mm i.d., 4um Gemini, RP18 column (Phenomenex-USA) was used for all analyses. The mobile phases were: A) water with 0.1% HCOOH and B) CH3CN. The following multi-step linear solvent gradient was used: from 0 to 5 min at 95% A; from 5 to 15 min to 75% A; then a plateau of 5 min, from 20 to 30 min to 20% A; then a plateau of 6 min; from 36 to 44 min at 5% A. The analysis was carried out for a total time of 30 min, plus an equilibration time of 10 min with flow rate 0.4 mL min^−1^, oven temperature 26 °C and injection volume 8 μL. The mass spectra were recorded in negative ion mode, setting the fragmentation energy between 80 and 180 V and applying the same chromatographic conditions as described previously. The mass spectrometer operating conditions were: gas temperature, 350 °C; nitrogen flow rate, 9 L min^−1^; nebulizer pressure, 30 psi; quadrupole temperature, 40 °C; and capillary voltage, 3500 V.

### 3.6. Amyloid Fibrils’ Preparation

HEWL was used as a well-established model to study amyloid fibril formation [30,55]. A HEWL stock solution (1 mM) was freshly prepared in 10 mM HCl (pH 2.0) and filtered through 0.2 μm filters (EMD Millipore, Milan, Italy). The formation of HEWL amyloid-type aggregates were obtained following the previously described method [29,30] by incubating the solution at 65 °C in a dry-bath (VWR International, Milan, Italy) for up to 10 days in static condition. Both HIE/m and HIE/n samples were mixed with HEWL at the same final 1:50 dilution, immediately before thermal incubation. HEWL amyloid aggregates grown in the presence of HIE/m or HIE/n are hereinafter called HEWL-HIE/m and HEWL-HIE/n, respectively. HEWL grown in the absence of HIEs was used as a control. The aggregation kinetics of HEWL was followed by the thioflavin T (ThT) fluorescence test [56]. Briefly, 195 μL of ThT (25 μM), freshly prepared in phosphate buffer (25 mM, pH 6.0), were added to 5 μL HEWL, HEWL-HIE/m or HEWL-HIE/n samples. ThT fluorescence values were recorded at an excitation/emission wavelengths of 440/485 nm using a FL Flouroskan Ascent microplate fluorimeter (Thermo Scientific, Waltham, MA, USA).

### 3.7. Atomic Force Microscopy

For Atomic Force Microscopy (AFM) analysis a drop of sample (diluted with water to 20 or 10 μM initial HEWL concentration) was vortexed and laid onto a freshly cleaved mica disc for about 2 min. Sample excess was removed by washing twice the sample mica disc with 1 mL of bi-distilled water, then the preparation was dried with a soft nitrogen flow. AFM experiments were performed in air, in non-contact mode, using a PicoSPM microscope equipped with an AAC-Mode controller (Agilent, USA, formerly Molecular Imaging, Phoenix, AZ, USA). The probes were non-contact Silicon cantilevers (model NSG-01, NT-MDT Co., Moscow, Russia) with 150 KHz typical resonance frequency. Scanner calibration was periodically checked by means of a reference grid (TGZ02 by MikroMash, Tallin, Estonia) with known 3 μm pitch and 100 nm step height. At least three different areas were imaged on each sample. Typical image size was in the range 1–10 μm, at 256 × 256 pixel resolution. The images were processed and analysed by WSxM software (Nanotec Electronica, Madrid, Spain), version 4.0 [43,57].

### 3.8. Cell Line

Human neuroblastoma SH-SY5Y cells, obtained from American Type Culture Collection (ATCC^®^, Manassas, VA, USA), were cultured in a 1:1 mixture of DMEM and Ham’s F12 supplemented with 2 mM L-glutamine, 100 μg/mL streptomycin, 100 U/mL penicillin and 10% FBS (complete medium), at 37 °C in a humidified atmosphere containing 5% CO_2_. At 90% confluence cells were appropriately propagated after trypsinization (trypsin 0.025%-EDTA 0.5 mM).

### 3.9. Cytotoxicity Evaluation

SH-SY5Y cell viability was assessed using the MTT assay adapted to cell line used in this work [43]. Briefly, cells were seeded in 96-well plate at a density of 5 × 10^3^ cells/well in complete medium overnight. For cytotoxicity experiments, 1:100 final dilutions of HEWL-HIE/m or HEWL-HIE/n aggregates were added to cells for 48 h. To exclude any possible contribution of the seaweed on cell viability, SH-SY5Y cells were also exposed to 0.11 and 1 μg/mL of HIE/m and HIE/n, respectively, corresponding to the amount of extract present in HEWL-HIE/m and HEWL-HIE/n samples added to cells. HEWL aggregates grown in the absence of HIEs were used as control. After cell treatment, cell medium was removed and MTT solution (0.5 mg/mL in PBS) was added into each well (100 μL/well). After 2 h incubation at 37 °C in the dark, cells were lysed with 100 μL/well of DMSO and the absorbance at 595 nm was measured using iMARK microplate reader (Bio-Rad, Philadelphia, PA, USA). Data were expressed as percentage ratio compared to untreated control cells.

### 3.10. Statistical Analysis

All the results were expressed in terms of mean ± standard deviation on the basis of at least three different experiment from three independent *H. incurva* extractions. Linear regression analysis on calibrator compounds and the corresponding interpolations were wade with Excel (Microsoft Corporation, Redmond, WA, USA). Non-linear curve fitting was performed with Origin 6.0 (OriginLab Corporation, Northampton, MA, USA).

## 4. Conclusions

The close relationship between the amyloid aggregation process and the onset of amyloidosis constantly encourages scientific research in the identification of new natural compounds capable of suppressing the formation of toxic amyloid aggregates. For the first time, our findings demonstrated the in vitro anti-amyloidogenic role of the *H. incurva*, whose metabolic composition and bioactivity were strongly influenced by seasonality. This work focused on the bioactivity of *H. incurva* phytocomplex to evaluate the synergistic action of its various constituents, while the structure and functionality of its secondary metabolites will be the subject of further studies. Overall, we believe that our findings can be a useful guide in relation to the screening of promising new natural anti-amyloidogenic agents in the search for innovative preventive strategies against neurodegeneration. Future investigations will aim to verify a potential in vitro disaggregating role of *H. incurva* on preformed amyloid fibrils and the possible cytoprotective role of the seaweed against amyloid aggregate-induced cytotoxicity, as well as its mechanism of action.

## Figures and Tables

**Table 1:** Final yield of hydroalcoholic extraction and biochemical composition of *H. incurva*. Values are reported as mean ± standard deviations from at least three independent experiments from three different extractions.

	HIE/m	HIE/n
Starting dry seaweed (g)	4	4
Total extract (mg)	58 ± 0.4	520 ± 41
Extraction yield (%)	1.45 ± 0.01	13 ± 1.02
	**mg/g respect to dry seaweed**	**mg/g respect to dry seaweed**
Total phenols *	1 ± 0.01	4.85 ± 0.1
Carbohydrates **	4.57 ± 0.45	10.48 ± 0.45
Antioxidant power ***	1.99 ± 0.21	3.22 ± 0.23
Radical scavenging ***	0.66 ± 0.05	10.73 ± 0.58
	**mg/g respect to dry extract**	**mg/g respect to dry extract**
Total phenols *	69.35 ± 1.13	37.34 ± 0.79
Carbohydrates **	315.34 ± 31.01	80.59 ± 3.84
Antioxidant power ***	137.39 ± 14.70	24.84 ±1.82
Radical scavenging ***	45.67 ± 3.62	82.57 ± 0.49

*: gallic acid, **: D-glucose, ***: ascorbic acid for standard calibration.

**Table 2:** Fragmentation pattern of the detected analytes in HIE/n extract applying a negative ionization mode and various fragmentors (80, 120 and 160 V).

Compound	Negative Ions (m/z)	Br Atoms (n°)
**3**	345; 347 [M-H]^–^; 267; 201; 199; 145; 102	1
**4**	345; 347 [M-H]^–^; 267; 201; 199; 145; 102	1
**5**	578; 576; 574 [M-H]^–^; 375; 373; 216; 214	-
**6**	578; 576; 574 [M-H]^–^; 373; 375; 216, 214	-
**8**	217; 215 [M-H]^–^; 135	-
**9**	549; 547; 545 [M-H]^–^; 465; 467; 401; 403; 345; 347; 319; 321; 211	2
**10**	549; 547; 545 [M-H]^–^; 465; 467; 401; 403; 345; 347; 319; 321; 211	2
**11**	549; 547; 545 [M-H]^–^; 465; 467; 401; 403; 345; 347; 319; 321; 211	2
**12**	958; 746; 211	-

## References

[1] Noble W., Burns M.P. (2010). Challenges in neurodegeneration research. Front. Psychiatry.

[2] Hussain R., Zubair H., Pursell S., Shahab M. (2018). Neurodegenerative Diseases: Regenerative Mechanisms and Novel Therapeutic Approaches. Brain Sci..

[3] Chiti F., Dobson C.M. (2006). Protein misfolding, functional amyloid, and human disease. Annu. Rev. Biochem..

[4] Currais A., Fischer W., Maher P., Schubert D. (2017). Intraneuronal protein aggregation as a trigger for inflammation and neurodegeneration in the aging brain. FASEB J..

[5] Giorgetti S., Greco C., Tortora P., Aprile F.A. (2018). Targeting Amyloid Aggregation: An Overview of Strategies and Mechanisms. Int. J. Mol. Sci..

[6] Stefani M., Rigacci S. (2013). Protein folding and aggregation into amyloid: The interference by natural phenolic compounds. Int. J. Mol. Sci..

[7] Haefner B. (2003). Drugs from the deep: Marine natural products as drug candidates. Drug Discov. Today.

[8] Russo P., Kisialiou A., Lamonaca P., Moroni R., Prinzi G., Fini M. (2016). New Drugs from Marine Organisms in Alzheimer’s Disease. Mar. Drugs.

[9] Bălașa A.F., Chircov C., Grumezescu A.M. (2020). Marine Biocompounds for Neuroprotection—A Review. Mar. Drugs.

[10] Pangestuti R., Kim S.K. (2011). Neuroprotective effects of marine algae. Mar. Drugs..

[11] Talarico L., Maranzana G. (2000). Light and adaptive responses in red macroalgae: An overview. J. Photochem. Photobiol. B.

[12] Cabrita M.T., Vale C., Rauter A.P. (2010). Halogenated compounds from marine algae. Mar. Drugs.

[13] De Nanteuil G., Mastagli P. (1981). A bromophenol in the red alga Halopitys incurvus. Phytochemistry.

[14] Chibi F., Rchid H., Arsalane W., Nmila R. (2018). Antioxidant activity and total phenolic content of the red alga Halopitys incurvus harvested from El Jadida Coast (Morocco). Int. J. Pharm. Phytochem. Res..

[15] Oumaskour K., Boujaber N., Etahiri S., Assobhei O. (2013). Anti-inflammatory and antimicrobial activities of twenty-three marine red algae from the coast of sidi bouzid (el jadida-morocco). Int. J. Pharm. Pharm. Sci..

[16] Spavieri J., Allmendinger A., Kaiser M., Itoe M.A., Blunden G., Mota M.M., Tasdemir D. (2013). Assessment of dual life stage antiplasmodial activity of british seaweeds. Mar. Drugs.

[17] Allmendinger A., Spavieri J., Kaiser M., Casey R., Hingley-Wilson S., Lalvani A., Guiry M., Blunden G., Tasdemir D. (2010). Antiprotozoal, antimycobacterial and cytotoxic potential of twenty-three British and Irish red algae. Phytother. Res..

[18] Díaz R.T.A., Chabrillón M., Cabello-Pasini A., Gómez-Pinchetti J.L., Figueroa F.L. (2010). Characterization of polysaccharides from Hypnea spinella (Gigartinales) and Halopithys incurva (Ceramiales) and their effect on RAW 264.7 macrophage activity. J. Appl. Phycol..

[19] Zbakh H., Salhi G., Moussa H., Riadi H. (2014). Cytotoxic and antioxidant activities of the red seaweed Halopithys incurva. Int. J. Adv. Pharm. Biol. Chem..

[20] Ngoungoure V.L., Schluesener J., Moundipa P.F., Schluesener H. (2015). Natural polyphenols binding to amyloid: A broad class of compounds to treat different human amyloid diseases. Mol. Nutr. Food Res..

[21] Olasehinde T.A., Olaniran A.O., Okoh A.I. (2019). Macroalgae as a Valuable Source of Naturally Occurring Bioactive Compounds for the Treatment of Alzheimer’s Disease. Mar. Drugs.

[22] Syad A.N., Devi K.P. (2015). Assessment of anti-amyloidogenic activity of marine red alga G. acerosa against Alzheimer’s beta-amyloid peptide 25–35. Neurol. Res..

[23] Leri M., Scuto M., Ontario M.L., Calabrese V., Calabrese E.J., Bucciantini M., Stefani M. (2020). Healthy Effects of Plant Polyphenols: Molecular Mechanisms. Int. J. Mol. Sci..

[24] Shariatizi S., Meratan A.A., Ghasemi A., Nemat-Gorgani M. (2015). Inhibition of amyloid fibrillation and cytotoxicity of lysozyme fibrillation products by polyphenols. Int. J. Biol. Macromol..

[25] Leri M., Nosi D., Natalello A., Porcari R., Ramazzotti M., Chiti F., Bellotti V., Doglia S.M., Stefani M., Bucciantini M. (2016). The polyphenol Oleuropein aglycone hinders the growth of toxic transthyretin amyloid assemblies. J. Nutr. Biochem..

[26] Mahdavimehr M., Meratan A.A., Ghobeh M., Ghasemi A., Saboury A.A., Nemat-Gorgani M. (2017). Inhibition of HEWL fibril formation by taxifolin: Mechanism of action. PLoS ONE.

[27] Merlini G., Bellotti V. (2005). Lysozyme: A paragmatic molecule for the investigation of protein structure, function and misfolding. Clin. Chim. Acta.

[28] Dumoulin M., Kumita J.R., Dobson C.M. (2006). Normal and aberrant biological self-assembly: Insights from studies of human lysozyme and its amyloidogenic variants. Acc. Chem. Res..

[29] Frare E., Polverino De Laureto P., Zurdo J., Dobson C.M., Fontana A. (2004). A highly amyloidogenic region of hen lysozyme. J. Mol. Biol..

[30] Ramazzotti M., Melani F., Marchi L., Mulinacci N., Gestri S., Tiribilli B., Degl’Innocenti D. (2016). Mechanisms for the inhibition of amyloid aggregation by small ligands. Biosci. Rep..

[31] Barletta E., Ramazzotti M., Fratianni F., Pessani D., Degl’Innocenti D. (2015). Hydrophilic extract from Posidonia oceanica inhibits activity and expression of gelatinases and prevents HT1080 human fibrosarcoma cell line invasion. Cell Adh. Migr..

[32] Ajisaka K., Agawa S., Nagumo S., Kurato K., Yokoyama T., Arai K., Miyazaki T. (2009). Evaluation and comparison of the antioxidative potency of various carbohydrates using different methods. J. Agric. Food Chem..

[33] Rocha de Souza M.C., Marques C.T., Guerra Dore C.M., Ferreira da Silva F.R., Oliveira Rocha H.A., Leite E.L. (2007). Antioxidant activities of sulfated polysaccharides from brown and red seaweeds. J. Appl. Phycol..

[34] Kladi M., Vagias C., Roussis V. (2004). Volatile halogenated metabolites from marine red algae. Phytochem. Rev..

[35] Pedersén M., Dasilva E.J. (1973). Simple Brominated Phenols in the Bluegreen Alga Calothrix brevissima West. Planta.

[36] Pedersén M., Saenger P., Fries L. (1974). Simple brominated phenols in red algae. Phytochemistry.

[37] Zhao J., Fan X., Wang S., Li S., Shang S., Yang Y., Xu N., Lü Y., Shi J. (2004). Bromophenol derivatives from the red alga Rhodomela confervoides. J. Nat. Prod..

[38] Buxbaum J.N., Linke R.P. (2012). A molecular history of the amyloidoses. J. Mol. Biol..

[39] Velander P., Wu L., Henderson F., Zhang S., Bevan D.R., Xu B. (2017). Natural product-based amyloid inhibitors. Biochem. Pharmacol..

[40] Koo E.H., Lansbury P.T., Kelly J.W. (1999). Amyloid diseases: Abnormal protein aggregation in neurodegeneration. Proc. Natl. Acad. Sci. USA.

[41] Hill S.E., Robinson J., Matthews G., Muschol M. (2009). Amyloid protofibrils of lysozyme nucleate and grow via oligomer fusion. Biophys. J..

[42] Hider R.C., Liu Z.D., Khodr H.H. (2001). Metal chelation of polyphenols. Method Enzymol..

[43] Ramazzotti M., Paoli P., Tiribilli B., Viglianisi C., Menichetti S., Degl’Innocenti D. (2017). Catechol-Containing Hydroxylated Biomimetic 4-Thiaflavanes as Inhibitors of Amyloid Aggregation. Biomimetics.

[44] Hudson S.A., Ecroyd H., Kee T.W., Carver J.A. (2009). The thioflavin T fluorescence assay for amyloid fibril detection can be biased by the presence of exogenous compounds. FEBS J..

[45] Choi D.Y., Lee Y.J., Hong J.T., Lee H.J. (2012). Antioxidant properties of natural polyphenols and their therapeutic potentials for Alzheimer’s disease. Brain Res. Bull..

[46] Devi K.P., Suganthy N., Kesika P., Pandian S.K. (2008). Bioprotective properties of seaweeds: In vitro evaluation of antioxidant activity and antimicrobial activity against food borne bacteria in relation to polyphenolic content. BMC Complement Altern. Med..

[47] Shanmuganathan B., Sheeja Malar D., Sathya S., Pandima Devi K. (2015). Antiaggregation Potential of Padina gymnospora against the Toxic Alzheimer’s Beta-Amyloid Peptide 25–35 and Cholinesterase Inhibitory Property of Its Bioactive Compounds. PLoS ONE.

[48] Stefani M. (2012). Structural features and cytotoxicity of amyloid oligomers: Implications in Alzheimer’s disease and other diseases with amyloid deposits. Prog. Neurobiol..

[49] Shaham-Niv S., Rehak P., Zaguri D., Levin A., Adler-Abramovich L., Vuković L., Král P., Gazit E. (2018). Differential inhibition of metabolite amyloid formation by generic fibrillation-modifying polyphenols. Comms. Chem..

[50] Leri M., Natalello A., Bruzzone E., Stefani M., Bucciantini M. (2019). Oleuropein aglycone and hy-droxytyrosol interfere differently with toxic Aβ1–42 aggregation. Food Chem. Toxicol..

[51] Palazzi L., Bruzzone E., Bisello G., Leri M., Stefani M., Bucciantini M., Polverino de Laureto P. (2018). Oleuropein aglycone stabilizes the monomeric α-synuclein and favours the growth of non-toxic aggregates. Sci. Rep..

[52] Rigacci S., Guidotti V., Bucciantini M., Nichino D., Relini A., Berti A., Stefani M. (2011). Aβ(1–42) aggregates into non-toxic amyloid assemblies in the presence of the natural polyphenol oleuropein aglycon. Curr. Alzheimer Res..

[53] Leri M., Ramazzotti M., Vasarri M., Peri S., Barletta E., Pretti C., Degl’Innocenti D. (2018). Bioactive Compounds from Posidonia oceanica (L.) Delile Impair Malignant Cell Migration through Autophagy Modulation. Mar. Drugs.

[54] Vasarri M., Leri M., Barletta E., Ramazzotti M., Marzocchini R., Degl’Innocenti D. (2020). Anti-inflammatory properties of the marine plant Posidonia oceanica (L.) Delile. J. Ethnopharmacol..

[55] Morozova-Roche L.A., Zurdo J., Spencer A., Noppe W., Receveur V., Archer D.B., Joniau M., Dobson C.M. (2000). Amyloid fibril formation and seeding by wild-type human lysozyme and its disease-related mutational variants. J. Struct. Biol..

[56] LeVine H. (1993). Thioflavine T interaction with synthetic Alzheimer’s disease beta-amyloid peptides: De-tection of amyloid aggregation in solution. Protein Sci..

[57] Horcas I., Fernández R., Gómez-Rodríguez J.M., Colchero J., Gómez-Herrero J., Baro A.M. (2007). WSXM: A software for scanning probe microscopy and a tool for nanotechnology. Rev. Sci. Instrum..

